# Regulatory mechanisms of simulated precipitation on cbbL carbon-fixing microbial communities in the alpine source wetland

**DOI:** 10.3389/fmicb.2025.1592315

**Published:** 2025-06-06

**Authors:** Ni Zhang, Ziwei Yang, Desheng Qi, Lin Li, Kelong Chen, Li Fu, Jianqing Sun

**Affiliations:** ^1^Qinghai Province Key Laboratory of Physical Geography and Environmental Process, College of Geographical Science, Qinghai Normal University, Xining, China; ^2^Key Laboratory of Tibetan Plateau Land Surface Processes and Ecological Conservation (Ministry of Education), Qinghai Normal University, Xining, China; ^3^National Positioning Observation and Research Station of Qinghai Lake Wetland Ecosystem in Qinghai, National Forestry and Grassland Administration, Haibei, China; ^4^College of Resources, Environment and Life Sciences, Ningxia Normal University, Guyuan, China, China; ^5^Qinghai Lake National Nature Reserve Administration, Xining, China

**Keywords:** Qinghai-Tibetan Plateau, Qinghai Lake, alpine wetland, carbon cycle, extreme precipitation

## Abstract

Global warming intensifies the hydrological processes in wetlands, thereby affecting the carbon dynamics of these ecosystems. The cbbL gene, a key gene involved in carbon fixation, is significantly influenced by changes in precipitation. In this study, precipitation manipulation treatments with 25 and 50% increases and decreases, along with a natural control, were established to assess the response of cbbL-carrying carbon-fixing microbial communities to altered precipitation in the source wetland of Qinghai Lake. Amplicon sequencing was conducted to characterize microbial community composition and dynamics. The results showed that with increased precipitation, the relative abundance of Actinobacteria exhibited a decreasing trend, while Cyanobacteria showed an increasing trend. Chlorophyta exhibited an “n”-shaped variation pattern (*P* < 0.05). Soil carbon and nitrogen were the most important factors influencing the cbbL carbon-fixing microbial community in the source wetland, with their concentrations decreasing as precipitation increased. The 25% increased precipitation treatment enhanced the environmental selection of cbbL carbon-fixing microbes, shifting the community assembly process from randomness to determinism. In addition, with the increase in precipitation, the network complexity and connectivity among cbbL carbon-fixing microbial species increased. In summary, reduced precipitation helps to enhance the carbon sequestration potential of the source wetland.

## 1 Introduction

Global warming has intensified many hydrological processes (IPCC, 2013), and these changes are likely to continue at an accelerated pace in the future (Bao et al., 2017; Gampe et al., 2021). Changes in precipitation patterns, such as the magnitude, frequency, and duration of rainfall, will threaten vulnerable terrestrial ecosystems as they interact with dynamic soil moisture changes (Fry et al., 2013). The carbon cycle, as a critical component of the Earth’s climate system, plays a vital role in understanding climate change and its impacts on ecosystems. Recent studies have assessed the effects of precipitation changes on soil carbon dynamics in terrestrial ecosystems. For example, Du et al. analyzed 248 published papers and concluded that soil respiration responses to precipitation changes are asymmetric, with soil carbon emissions expected to decrease under future precipitation regimes (Du et al., 2020). Chen et al. studied the effects of precipitation on soil carbon dynamics and found that under increased precipitation conditions, the concentrations of soluble organic carbon and microbial biomass carbon in wetland soils significantly decreased (Chen et al., 2023). Wetland ecosystems, as key components of the global carbon cycle, are significantly influenced by hydrological processes, which affect soil nutrients and carbon content (Mitsch and Mander, 2018; Feng et al., 2020). In recent years, precipitation has generally been increasing at a rate of 2.2% per decade. It is projected that in the near term (current–2050) and long term (2051–2100), precipitation will increase by 10.4–11% and 14.2–21.4%, respectively, compared to the baseline period (Chen et al., 2015). These changes will lead to dramatic shifts in wetland carbon dynamics.

Soil microorganisms dominate the soil nutrient mineralization process (Nottingham et al., 2017) and are crucial for terrestrial carbon (C) balance and C-climate feedback (Chapin and Vitousek, 2011; Paul, 2015). Increased precipitation generally favors microbial growth and soil C storage processes (Raczka et al., 2022; Nielsen and Ball, 2015). Soil auto-trophic bacteria play a key role in soil carbon sequestration, and their abundance is primarily driven by the mean annual precipitation (Selesi et al., 2005; Zhao et al., 2018). Most of these bacteria utilize atmospheric CO_2_ through the Calvin-Benson-Bassham (CBB) cycle, with the cbbL gene, which encodes a large subunit of RubisCO I, frequently used as a phylogenetic marker to study autotrophic bacterial communities (Kovaleva et al., 2011). Therefore, exploring the response mechanisms of the cbbL carbon-fixing microbial community in wetlands under precipitation changes is of significant theoretical and practical value for understanding the carbon sequestration processes in wetlands and their environmental regulatory roles.

The Qinghai-Tibet Plateau is the region most affected by the uncertainty of environ-mental changes due to global climate change (Tandong, 2019) and also has the largest area of alpine wetlands in the world (Bai et al., 2015). Changes in various hydrological processes in this region may have adverse effects on its ecological structure, function, and resilience (McLauchlan et al., 2013; O’Beirne et al., 2017). The Qinghai Lake Basin is a high-latitude internationally important wetland. As a major distribution area of wetlands on the Qinghai–Tibet Plateau, the region exhibits climatic heterogeneity, making its wetlands particularly unique (Cui and Li, 2015; Li et al., 2019; Qi, 2012). Therefore, this study focuses on the source wetland in the Qinghai Lake Basin. High-throughput sequencing technology was used to assess the microbial community of the cbbL function-al gene, while the biogeochemical properties of the soil were also measured. This study aims to address the following questions: (1) Investigate the response patterns of the cbbL carbon-fixing microbial community in alpine source wetlands to different precipitation gradients; (2) Examine the impact of precipitation-driven soil properties on the cbbL carbon-fixing microbial community in source wetlands; (3) Analyze the community assembly process and microbial interaction network changes of cbbL carbon-fixing microbes under different precipitation gradients in source wetlands. By comparing the responses of carbon-fixing microbial communities under varying precipitation conditions, new insights into wetland carbon sequestration mechanisms can be provided, thereby offering scientific guidance for the management of wetland ecosystems under global climate change.

## 2 Materials and methods

### 2.1 Study site and soil sampling

Qinghai Lake is located in the northeastern part of the Qinghai-Tibet Plateau, where precipitation is concentrated from May to September, and the region experiences a distinct plateau continental climate. The study area is situated at the headwaters of the Shaliu River on the northern shore of Qinghai Lake, near Wayan Mountain (37°43′∼37°46′N, 100°01′∼100°05′E), with significant diurnal temperature variation. The long-term average annual temperature is –3.31°C, and the long-term average annual precipitation is 420.37 mm. The vegetation in this area is relatively simple, dominated by Kobresia humilis. The plots were established in 2018, with a total of 15 plots, grouped into five treatments of three plots each, named as follows: Wck (natural control), WZa (50% increased precipitation), WZb (25% increased precipitation), WJa (50% decreased precipitation), and WJb (25% decreased precipitation). Precipitation reduction was achieved by covering the corresponding area with equidistantly inclined diversion channels, while precipitation in-crease was achieved by spraying water into the ground through pipes that diverted water into the channels designed for reduced precipitation. In June 2020, five soil cores (diameter 4.5 cm, depth 0–10 cm) were randomly collected from each plot and mixed to form a composite soil sample. Visible roots and other plant residues were removed, and all soil samples were sieved through a 2 mm mesh. One portion of the samples was stored at 4°C for the analysis of soil carbon, nitrogen content, and pH. The other portion was stored at –80°C for high-throughput sequencing of soil carbon-fixing microbes.

### 2.2 Determination of soil physical and chemical properties

The elemental analyzer (Vario EL III, Elemental Analysis System GmbH, Germany) measured the total carbon and total nitrogen contents of the soil. Soil pH was measured using a pH meter (FE20-FiveEasy pH, Mettler Toledo, Germany) with a soil-to-water ratio of 1:2.5. Soil moisture was monitored using a TDR-300 (Spectrum Technologies, Plainfield, Illinois, United States), and soil temperature was measured using a LI-8100 system (LI-COR, Lin-coln, Nebraska, United States).

### 2.3 DNA extraction and cbbL gene amplification

Microbial DNA was extracted from soil using the PowerSoil DNA Isolation Kit (Mo Bio, Carlsbad, CA, United States), and the purity of the DNA was assessed using a NanoDrop2000 UV-Vis spectrophotometer (Thermo Scientific, Wilmington, DE, United States). The cbbL gene fragment was amplified using a thermal cycler PCR (Polymerase Chain Reaction) system (GeneAmp 9700, ABI, United States) with the primers (5′-GACTTCACCAAAGACGACGA-3′) and (5′-TCGAACTTGATTTCTTTCCA-3′). The purified amplicons were pooled in equal amounts and sequenced on the Illumina MiSeq platform (San Diego, United States).

### 2.4 Statistical analysis

The MicrobiotaProcess package was used to calculate the diversity of the carbon-fixing microbial communities in different groups. Non-metric multidimensional scaling (NMDS), non-parametric multivariate analysis of variance (ADONIS), and non-parametric tests (ANOSIM) were applied to analyze the differences in community structure between samples based on Bray-Curtis dissimilarity. The UpSetR package was used to visualize shared and unique OTUs (Operational Taxonomic Units) between groups, and the rdacca.hp package quantified the relative influence of soil physicochemical factors on changes in the soil carbon-fixing microbial community using hierarchical partitioning modeling. The FAPROTAX database was used to predict ecological functions related to biogeochemical cycles (Liang et al., 2020), to analyze the impact of different precipitation gradients on the functional groups of soil carbon-fixing microbes. The significance of differences was tested using one-way analysis of variance (ANOVA) (*P* < 0.05). The betaNTI index was calculated using the Picante package based on null models, and the Raup-Crick (RCbray) index was computed with the microeco package to analyze the impact of different precipitation gradients on the assembly of carbon-fixing microbial communities. The psych package was used to calculate correlations between the data, and network diagrams were visualized using Gephi 0.9.7. All R packages were run in R software (v4.1.2).

## 3 Results

### 3.1 Response of soil physicochemical properties to precipitation changes

**FIGURE 1:**
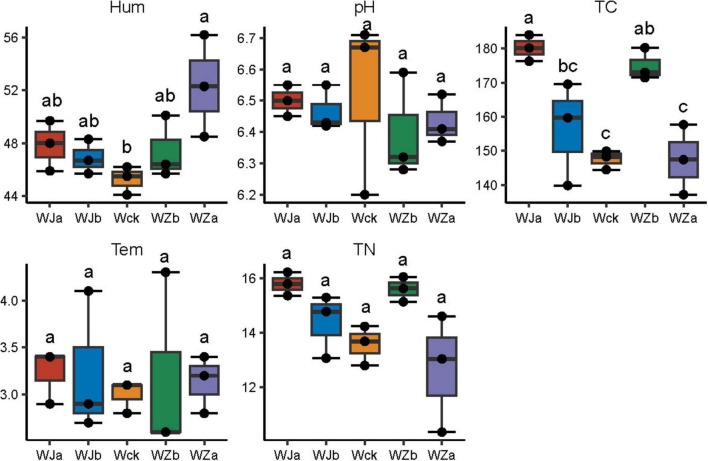
Physicochemical properties of soil under precipitation changes in the source wetland. Letters a-c indicates significance, with the same letter representing no significant difference (*P* > 0.05) and different letters indicating a significant difference (*P* < 0.05). Tem, soil temperature; Hum, soil humidity; TN, total nitrogen; TC, total carbon; pH, soil pH.

The soil microenvironment in the source wetland was regulated by precipitation changes, exhibiting distinct physicochemical properties (Figure 1). Soil moisture ranged from 44.1 to 56.2%, with a significant increase in soil moisture under the 50% increased precipitation treatment (*P* < 0.05, Figure 1). The total soil carbon content ranged from 137.2 to 184.0 g/kg, with significant increases in total carbon under the 50% decreased and 25% increased precipitation treatments (*P* < 0.05, Figure 1). Soil pH in the source wetland ranged from 6.2 to 6.7, with temperatures ranging from 2.6 to 4.3°C. The trend in total nitrogen content mirrored that of total carbon, ranging from 10.4 to 16.2 g/kg, though no statistically significant differences were observed (*P* > 0.05, Figure 1).

### 3.2 Response of the cbbL carbon-fixing microbial community structure to precipitation changes

**FIGURE 2:**
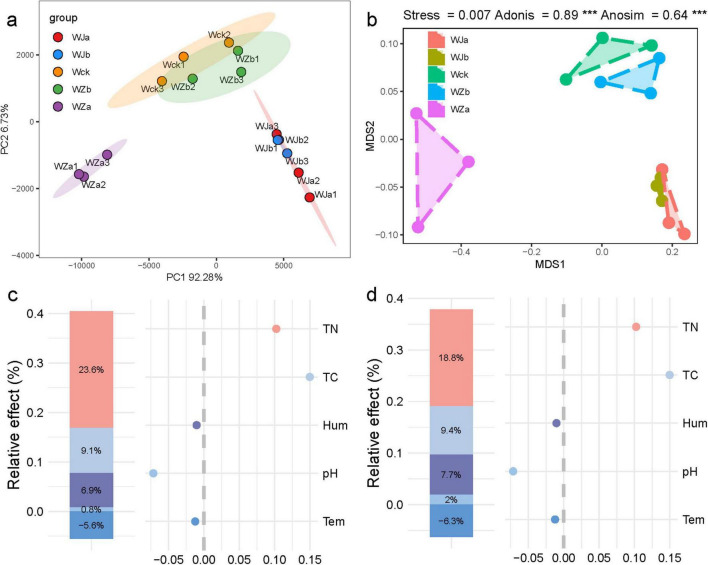
Diversity of carbon-fixing microbial communities and factors influencing community structure in the source wetland. **(a)** PCA principal component analysis; **(b)** validation of group effectiveness; **(c)** hierarchical partitioning analysis of factors influencing carbon-fixing microbial community structure; **(d)** hierarchical partitioning analysis of factors influencing carbon-fixing microbial community diversity. Tem, soil temperature; Hum, soil humidity; TN, total nitrogen; TC, total carbon; pH, soil pH.

To compare the effects of precipitation changes on the diversity of the cbbL carbon-fixing microbial community in the source wetland, the ACE index, Chao1 index, Shannon index, and Simpson index were calculated. The results showed that precipitation changes did not significantly alter the community diversity of cbbL carbon-fixing microbes in the source wetland, but the response patterns of community diversity to different precipitation gradients were different (*P* > 0.05, Supplementary Figure 1). Under the 50% increased precipitation treatment, the community diversity indices increased, while the opposite was observed under the 25% increased precipitation treatment. Furthermore, the precipitation reduction treatments increased the richness indices (ACE, Chao1), but decreased the diversity indices (Shannon, Simpson; *P* > 0.05, Supplementary Figure 1). The total explained variance of the PCA analysis reached 99.01%, and the precipitation treatments strongly altered the cbbL carbon-fixing microbial community in the source wetland, forming unique groups distinct from the control soils (Figure 2a). Results from NMDS, Adonis, and ANOSIM analyses showed significant differences between the groups, confirming the PCA results (*P* < 0.05, Figure 2b). Hierarchical partitioning analysis revealed that soil carbon and nitrogen con-tent were the most important explanatory factors for changes in the cbbL carbon-fixing microbial community structure and diversity (Figures 2c,d), explaining 23.6 and 18.8% of the community structure variation, respectively. Soil moisture also had some influence on community characteristics, while temperature showed a negative effect on community traits (Figures 2c,d).

**FIGURE 3:**
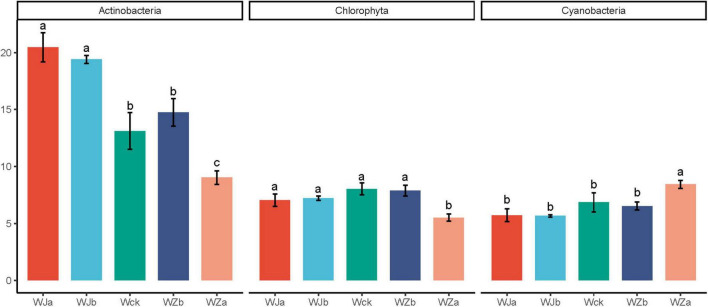
Phylum-level differential microbial communities under different treatments in the source wetland. Letters a-c indicates significance, with the same letter representing no significant difference (*P* > 0.05) and different letters indicating a significant difference (*P* < 0.05).

**FIGURE 4:**
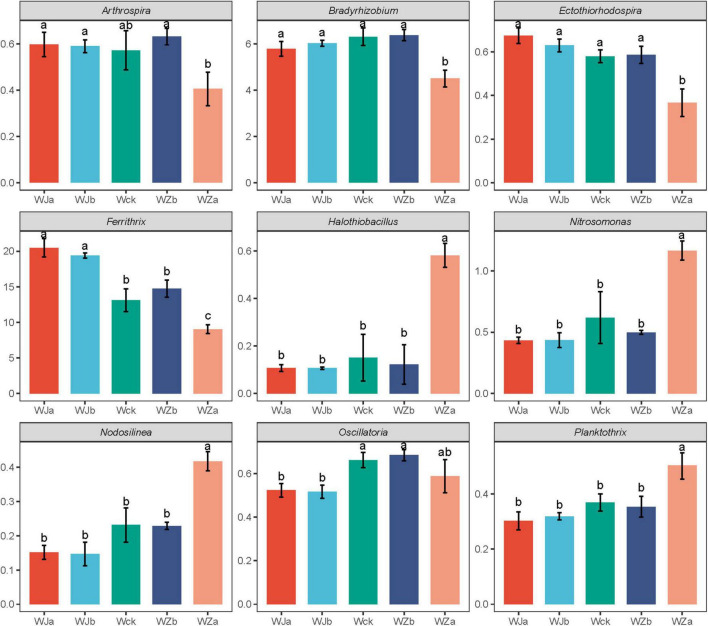
Genus-level differential microbial communities under different treatments in the Lakeside Wetland. Letters a-c indicates significance, with the same letter representing no significant difference (*P* > 0.05) and different letters indicating a significant difference (*P* < 0.05).

An OTU-based UpSet plot analysis was used to measure the overlap between cbbL microbial communities (Supplementary Figure 2). The total number of shared OTUs across all groups was 793, with the total number of OTUs for WZa, WZb, Wck, WJa, and WJb being 1170, 1022, 1057, 1047, and 1075, respectively. The number of unique OTUs for these groups was 78, 13, 13, 18, and 22, with the WZa treatment showing the highest total and unique OTU numbers. Supplementary Figure 3 shows the phylum-level community composition of cbbL carbon-fixing microbes in the source wetland. The dominant phyla were Proteobacteria (61.49–63.99%), Actinobacteria (8.60–21.65%), Cyanobacteria (5.31–8.80%), and Chlorophyta (5.26–8.42%). Among them, the relative abundance of Actinobacteria showed a de-creasing trend with increasing precipitation, while precipitation reduction treatments significantly increased the relative abundance of this phylum. The 50% increased precipitation treatment significantly reduced its relative abundance (*P* < 0.05, Figure 3). Chlorophyta exhibited an “n”-shaped pattern, with the 50% increased precipitation treatment significantly reducing its relative abundance (*P* < 0.05, Figure 3). Cyanobacteria showed an increasing trend, with the 50% increased precipitation treatment significantly increasing its relative abundance (*P* < 0.05, Figure 3). The dominant genera of cbbL carbon-fixing microbes in the source wetland were 10 in total, with *Thioflexothrix* (19.29%) and *Ferrithrix* (15.35%) having relatively high abundances (Supplementary Figure 4). ANOVA test results showed that precipitation treatments significantly altered the relative abundances of nine of these genera, excluding *Thioflexothrix*. Specifically, the relative abundances of *Halothiobacillus*, *Nitrosomonas*, *Nodosilinea*, and *Planktothrix* increased with increasing precipitation, with the 50% increased precipitation treatment significantly enhancing their relative abundances. In contrast, the relative abundances of *Ferrithrix* and *Ectothiorhodospira* showed the oppo-site trend, with the 50% increased precipitation treatment significantly reducing their relative abundances, while the precipitation reduction treatment significantly increased the relative abundance of *Ferrithrix*. The relative abundances of *Arthrospira*, *Bradyrhizobium*, and Oscillatoria exhibited an “n”-shaped pattern with increasing precipitation, with the 50% increased precipitation treatment significantly reducing the relative abundances of *Arthrospira* and *Bradyrhizobium*, and the precipitation reduction treatment significantly reducing the relative abundance of *Oscillatoria* (*P* < 0.05, Figure 4).

### 3.3 Community assembly mechanisms and functional groups of cbbL carbon-fixing microbes in response to precipitation changes

**FIGURE 5:**
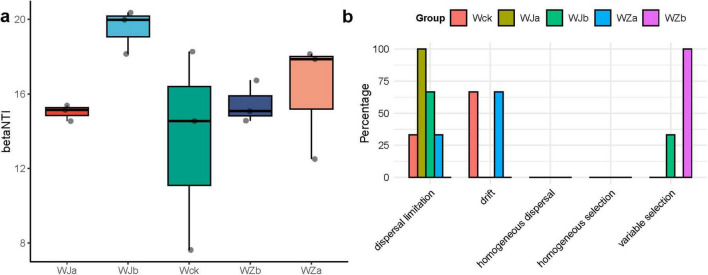
Community assembly of cbbL carbon-fixing microbes in the source wetland. **(a)** Distribution of the βNTI index of carbon-fixing microbial communities. **(b)** Distribution of the community assembly processes of carbon-fixing microbes.

Both deterministic and stochastic processes jointly drove the community assembly of cbbL carbon-fixing microbes in the source wetland. The calculation of the community assembly βNTI values indicated that, at the regional scale, deterministic processes dominated the community assembly of carbon-fixing microbes under different precipitation treatments (|βNTI| > 2) (Figure 5a). Further calculation of RCbray was performed to differentiate the relative contributions of diffusion limitation, drift, homogeneous diffusion, and selection to community changes (Figure 5b). The results indicated that during the community assembly of carbon-fixing microbes in the Qinghai Lake Wetland, the relative contributions of deterministic and stochastic processes under different precipitation treatments changed. Diffusion limitation dominated the community assembly process under the precipitation reduction treatment, while drift dominated the community assembly under the natural control and 50% increased precipitation treatments. The community assembly under the 25% increased precipitation treatment was driven by heterogeneous selection (Figure 5b). Additionally, both the natural control and 50% increased precipitation treatments showed a certain degree of diffusion limitation, accounting for 33.3%, while the 25% precipitation reduction treatment showed a certain degree of heterogeneous selection (33.3%, Figure 5b).

**FIGURE 6:**
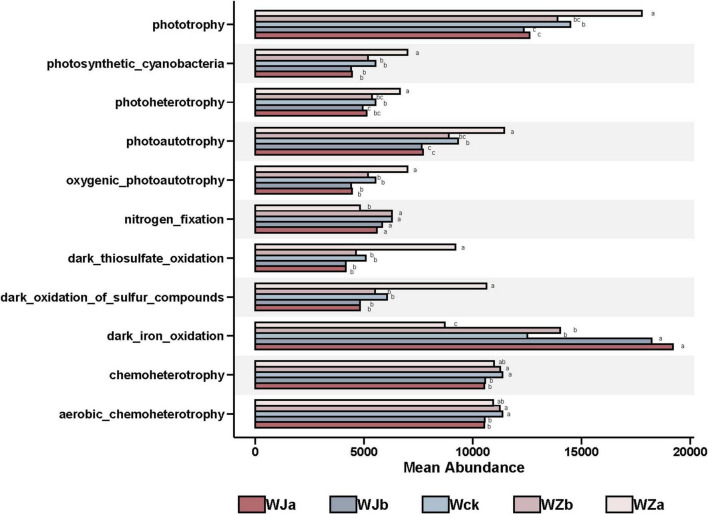
Differential functional groups under different treatments in the source wetland. Letters a-c indicates significance, with the same letter representing no significant difference (*P* > 0.05) and different letters indicating a significant difference (*P* < 0.05).

The predicted functional groups of carbon-fixing microbes (Supplementary Figure 5) showed that their functional categories were primarily focused on dark iron oxidation (10.74%), phototrophy (10.52%), photoautotrophy (8.09%), chemoheterotrophy (8.08%), aerobic chemo-heterotrophs (6.66%), dark oxidation of sulfur compounds (4.71%), dark thiosulfate oxidation (4.27%), photosynthetic cyanobacteria (4.09%), oxygenic photoautotrophy (4.03%), photoheterotrophy (3.39%), and nitrogen fixation (3.39%), accounting for over 67% of the total. Precipitation treatments significantly altered the relative abundance of 11 functional groups. The relative abundances of nitrogen fixation, aerobic chemoheterotrophy, and chemoheterotrophy exhibited an “n”-shaped response to increasing precipitation, while the relative abundance of dark iron oxidation decreased with increasing precipitation. The relative abundance of the remaining seven functional groups increased with increasing precipitation (*P* < 0.05, Figure 6).

#### 3.4 Response of the co-occurrence network of cbbL carbon-fixing microbes to precipitation changes

**FIGURE 7:**
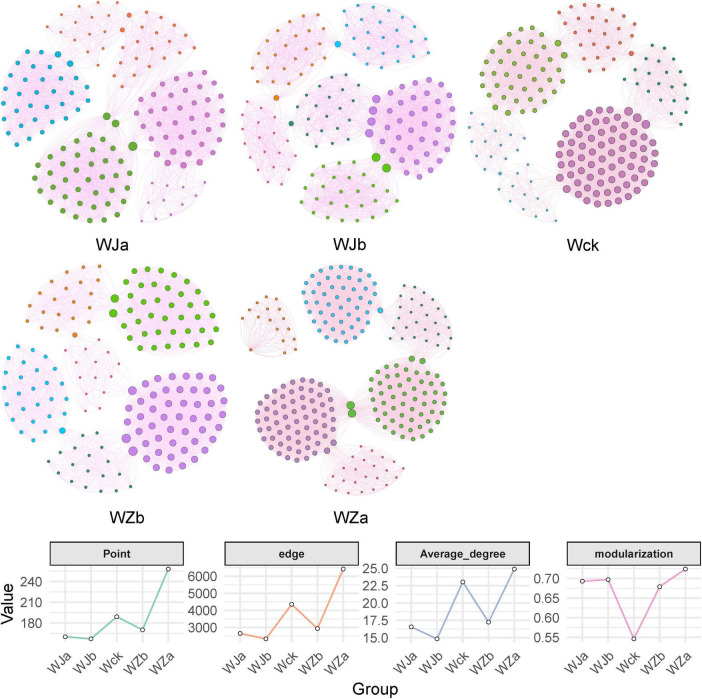
Network patterns **(a)** and key topological properties **(b)** of cbbL carbon-fixing microbes under different treatments in the source wetland. The size of the nodes represents the degree. Node colors represent different modules. Edge colors indicate positive or negative correlations, with red representing positive correlations and green representing negative correlations.

The results of the carbon-fixing microbial network analysis under different precipitation treatments showed varying trends in the topological properties (Figure 7 and Table 1). The network complexity and connectivity between species of cbbL carbon-fixing microbes both increased with precipitation, reaching their highest values under the + 50% precipitation treatment, with the average degree following a similar trend (Figure 7 and Table 1). Additionally, the modularity of the network exhibited a V-shaped response to increasing precipitation, with both the increased and decreased precipitation treatments enhancing the modularity of the microbial network (Figure 7 and Table 1).

**TABLE 1:** Topological parameters of the cbbL carbon-fixing microbial network under precipitation variation in the source wetland.

Group	Average degree	Modularization	Edge	Negative correlation	Positive correlation	Point
WJa	16.55	0.693	2648	160	WJa	16.55
WJb	14.828	0.697	2328	157	WJb	14.828
Wck	23.032	0.546	4353	189	Wck	23.032
WZb	17.235	0.679	2930	170	WZb	17.235
WZa	24.891	0.724	6422	258	WZa	24.891

## 4 Discussion

### 4.1 Response of cbbL carbon-fixing microbial communities to precipitation changes in the source wetland

Microbial communities in alpine wetland ecosystems exhibit different response strategies to varying precipitation gradients (Li et al., 2024). Moderate soil moisture can promote the α-diversity of cbbL bacteria (He et al., 2024). In this study, the 50% increased precipitation treatment in the source wetland increased the diversity index of carbon-fixing microbial communities. It was also found that both the 25% increased and reduced precipitation treatments led to a decrease in the diversity index, further confirming that the negative impact of reduced precipitation on microbial diversity outweighs the positive effect of increased precipitation (Yang et al., 2021). However, precipitation changes did not significantly alter the cbbL carbon-fixing microbial community diversity in the source wetland, which is consistent with the findings of Li et al. They analyzed the microbial community diversity under seven precipitation gradients and also found no significant difference in the α-diversity index (Li X. et al., 2022).

The dominant carbon-fixing microbial phyla at the phylum level in the source wetland were not significantly affected by the precipitation gradient, with Proteobacteria being the most abundant and the dominant phylum in the soil. This is consistent with previous studies (Li X. et al., 2022). The dominant genera of carbon-fixing microorganisms in the source wetland were *Thioflexothrix* and *Ferrithrix*, which differs from previous conclusions. Wang et al. found that the dominant genera of carbon-fixing microorganisms in karst wetlands were *Rubrivivax*, *Cyanobium*, and *Methyllium* (Wang X. et al., 2022). Recent studies on estuarine carbon-fixing bacteria indicated that the dominant genus of carbon-fixing bacteria containing the cbbL gene was *Endothiovibrio* (Xu et al., 2024). This difference is likely due to the distinct soil properties and genetic characteristics determined by different study regions, leading to variations in the dominant populations of carbon-fixing microorganisms (Tolli and King, 2005).

In the source wetland, some microbial communities were highly sensitive to precipitation changes. The relative abundance of Actinobacteria decreased with increasing precipitation, and *Ferrithrix* exhibited a similar trend. This may be because they have a higher tolerance to water stress, allowing them to maintain their abundance better in drier environments (Evans and Wallenstein, 2014; Ochoa-Hueso et al., 2018). Cyanobacteria and Chlorophyta are capable of fixing CO_2_ through photosynthesis using light energy (Nguyen et al., 2021). The relative abundance of the former increased with precipitation, likely responding to changes in unstable carbon availability caused by precipitation variations (Fierer et al., 2009). The relative abundance of the latter exhibited an n-shaped response, which may be due to the negative effects of insufficient precipitation on microbial activity (Henry, 2013), and excessive precipitation affecting photosynthesis, hindering microbial growth and reproduction.

### 4.2 Influencing factors, community construction, and interrelationships of cbbL carbon-fixing microorganisms in the source wetland

Precipitation changes in the source wetland altered soil moisture (Zhang et al., 2019), leading to significant changes in soil total carbon content. Previous studies have shown that changes in soil carbon and nitrogen can significantly affect the carbon fixation rate of soil microorganisms, thereby altering the microbial carbon fixation process (Li et al., 2021). Our study also found that soil carbon and nitrogen content was the most important explanatory factor for the changes in the cbbL carbon-fixing microbial community structure and diversity (Lynn et al., 2017; Wang J. et al., 2022). Both deterministic and stochastic processes jointly drove the construction of the cbbL carbon-fixing microbial community in the source wetland. With increasing precipitation, the microbial community construction shifted from stochastic to deterministic processes (under the 25% rainfall increase treatment), and then back to stochastic processes (under the 50% rainfall increase treatment). This finding is consistent with recent studies (Du et al., 2023). Furthermore, the 25% rainfall increase treatment enhanced the environmental selection effect on carbon-fixing microorganisms.

Microorganisms do not exist in isolation but form a complex network of relationships (Faust and Raes, 2012). In the source wetland, the cbbL carbon-fixing microbial community showed tighter community connections under the rainfall increase treatments, while the connections were more distant under the rainfall decrease treatments (Hu et al., 2022). This suggests that increased precipitation may enhance the synergistic or competitive interactions between different bacterial species (He et al., 2017). Both the increased and decreased rainfall treatments increased the modularity of the cbbL carbon-fixing microbial network, which may be due to reduced environmental stress caused by precipitation changes, leading to an increase in microbial community modularity (Hernandez et al., 2021). Microbial interactions are considered an important driving force for ecosystem functions (Allen et al., 2022). The seven main functional groups of carbon-fixing microorganisms in the source wetland showed an increasing trend with increasing precipitation, which was highly consistent with the changes in the microbial interaction network. This indicates that increased precipitation is beneficial for improving the functional potential of soil cbbL carbon-fixing microorganisms (Chen et al., 2022; Cotrufo et al., 2013).

## 5 Conclusion

This study investigated the effects of precipitation changes on the soil cbbL carbon-fixing microbial community and soil physicochemical properties in the source wetland of Qinghai Lake. Increased precipitation was found to reduce the carbon sequestration potential of the source wetland, enhance synergistic interactions among carbon-fixing microorganisms, and improve the functional potential of soil cbbL carbon-fixing microbial communities. Additionally, the 25% increased precipitation treatment enhanced the environmental selection of the cbbL carbon-fixing microorganisms in the source wetland. Soil carbon and nitrogen content was identified as the most important explanatory factor for changes in the microbial community characteristics. These findings advance our understanding of the potential mechanisms of carbon storage in high-altitude source wetlands and contribute to promoting effective ecosystem management.

## Data Availability

The datasets presented in this study can be found in online repositories. The names of the repository/repositories and accession number(s) can be found below: https://www.ncbi.nlm.nih.gov/, PRJNA1210473.

## Supplementary material

Supplementary Figure 1 Alpha diversity index. “NS” indicates *P* > 0.05.

Supplementary Figure 2 Distribution of OTUs.

Supplementary Figure 3 Dominant phyla.

Supplementary Figure 4 Dominant genera.

Supplementary Figure 5 Functional groups.
