# Supplementary figures and images for: Regulatory mechanisms of simulated precipitation on cbbL carbon-fixing microbial communities in the alpine source wetland
Source: Front Microbiol. 2025 Jun 6;16:1592315. doi: 10.3389/fmicb.2025.1592315 (PMC12179155; doi:10.3389/fmicb.2025.1592315)

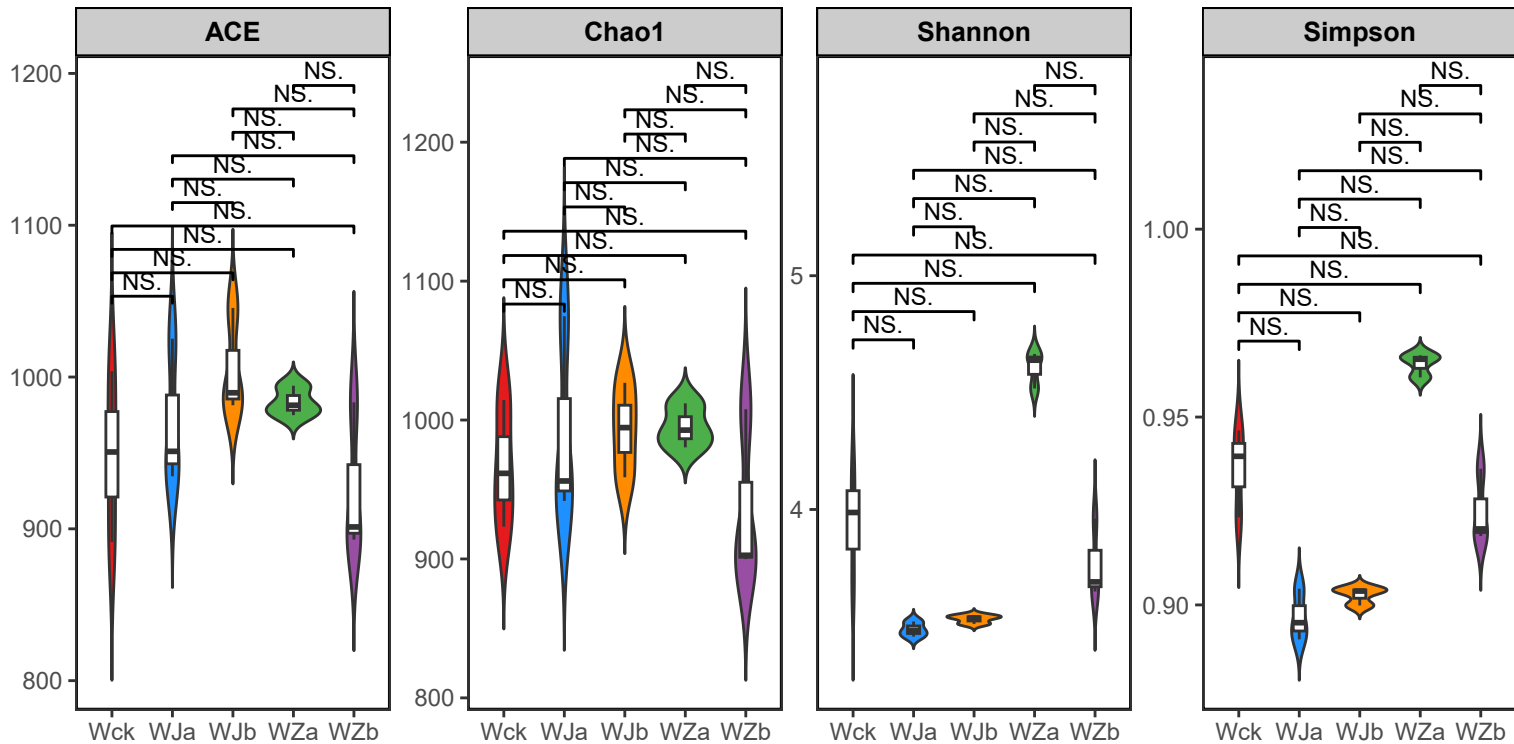

Supplement: Supplementary Figure 1 — Alpha diversity index. “NS” indicates P > 0.05. [file Data_Sheet_1.pdf]

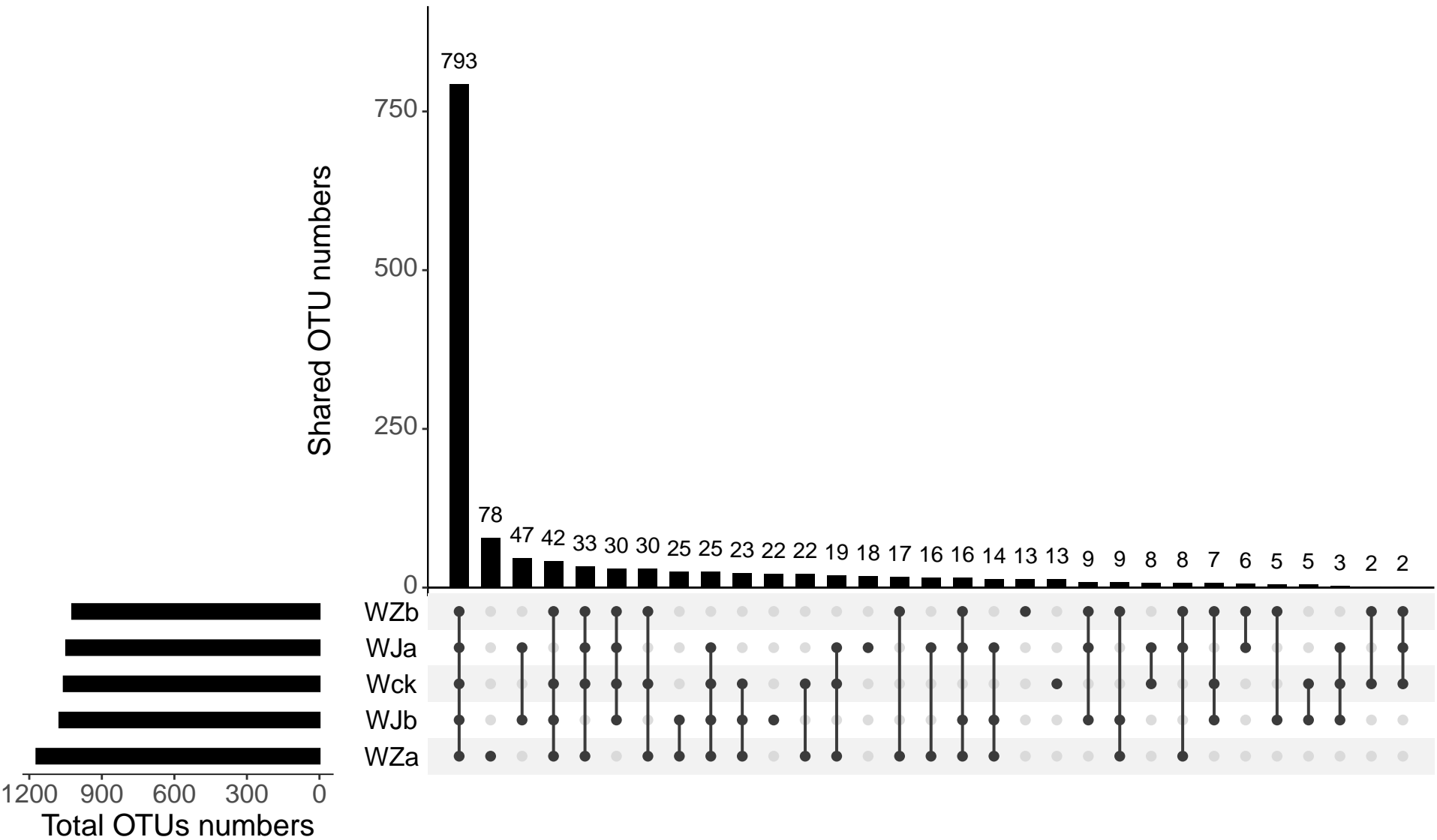

Supplement: Supplementary Figure 2 — Distribution of OTUs. [file Data_Sheet_2.pdf]

Chlorophyta Cyanobacteria Actinobacteria Proteobacteria Other

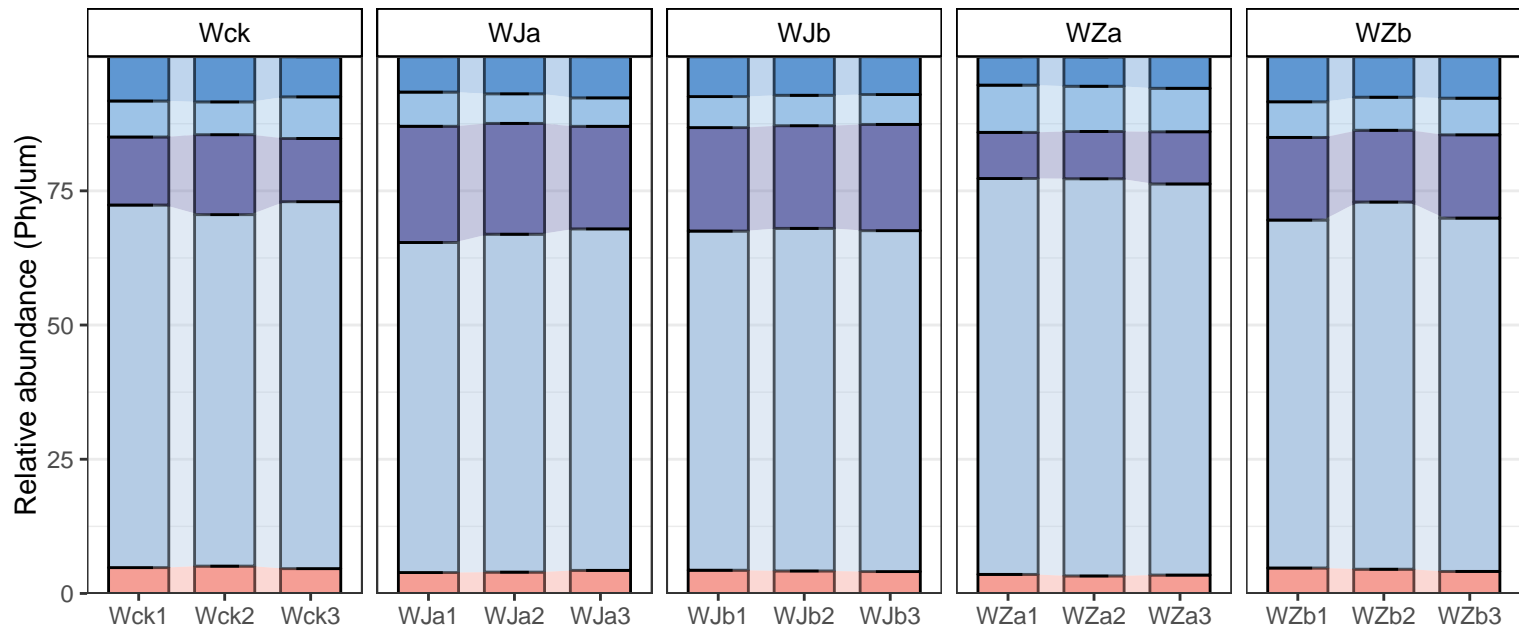

Supplement: Supplementary Figure 3 — Dominant phyla. [file Data_Sheet_3.pdf]

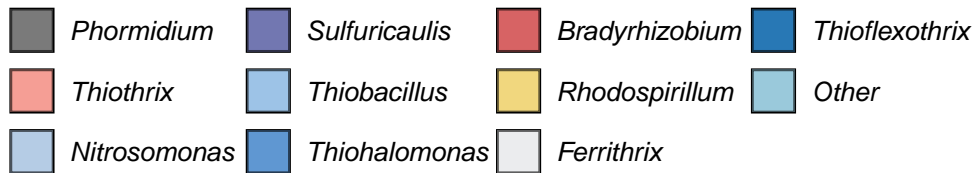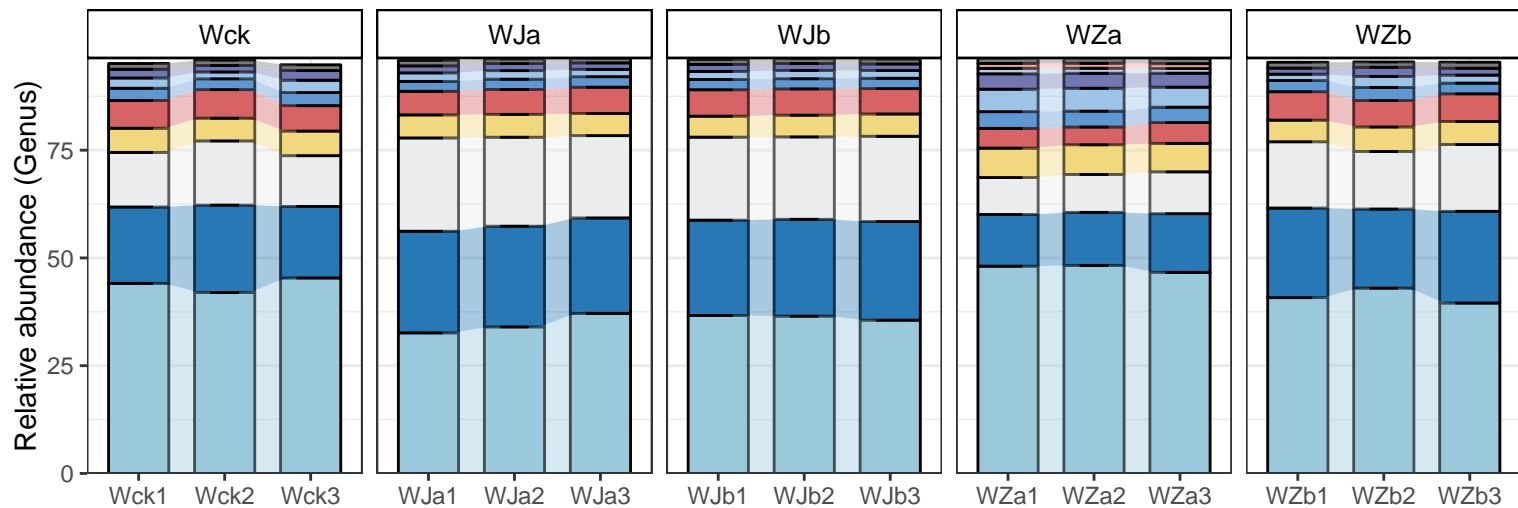

Supplement: Supplementary Figure 4 — Dominant genera. [file Data_Sheet_4.pdf]

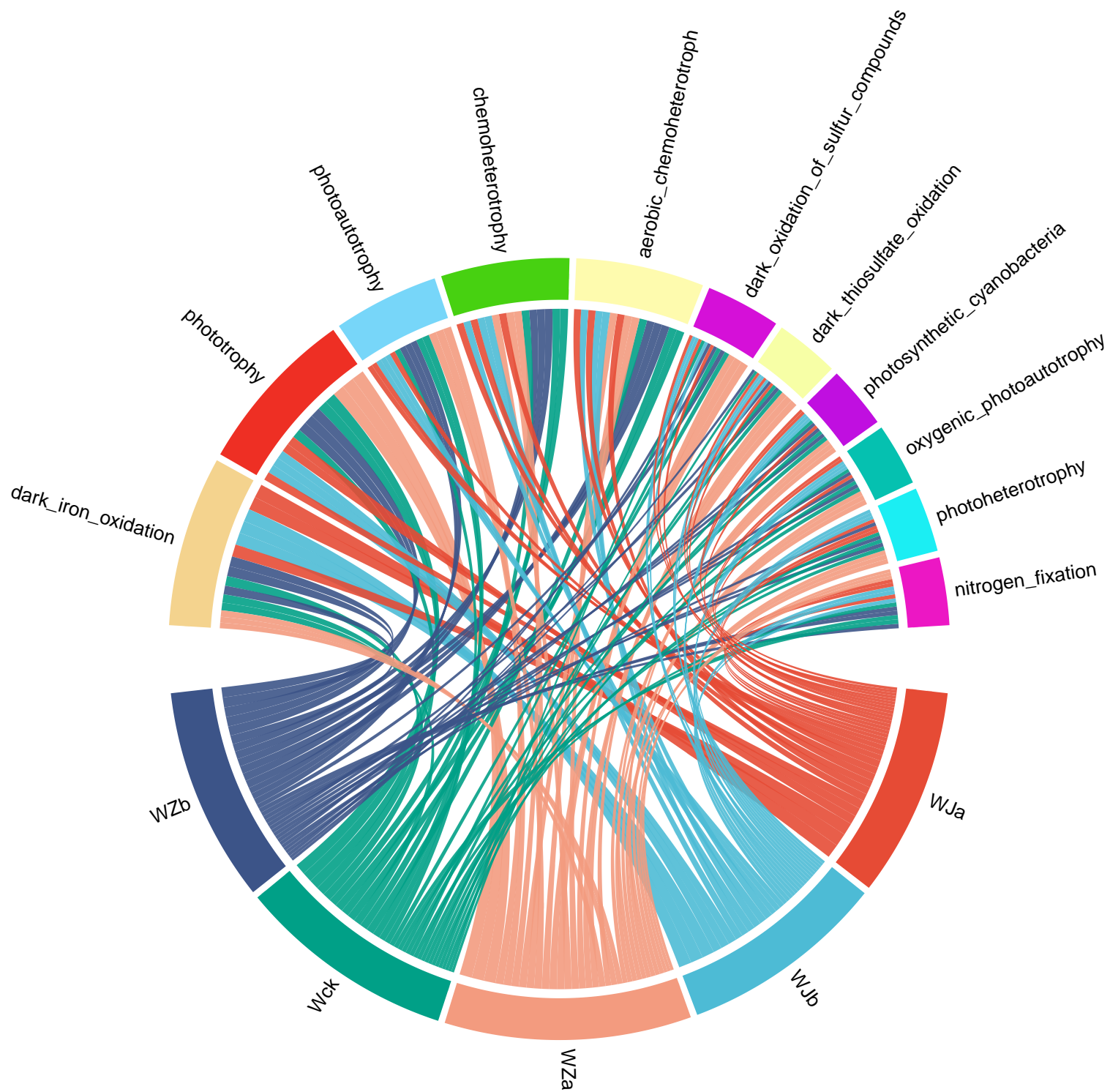

Supplement: Supplementary Figure 5 — Functional groups. [file Data_Sheet_5.pdf]
